# 50 Years of *Emmonsia* Disease in Humans: The Dramatic Emergence of a Cluster of Novel Fungal Pathogens

**DOI:** 10.1371/journal.ppat.1005198

**Published:** 2015-11-19

**Authors:** Ilan S. Schwartz, Chris Kenyon, Peiying Feng, Nelesh P. Govender, Karolina Dukik, Lynne Sigler, Yanping Jiang, J. Benjamin Stielow, José F. Muñoz, Christina A. Cuomo, Alfred Botha, Alberto M. Stchigel, G. Sybren de Hoog

**Affiliations:** 1 International Health Unit, Department of Epidemiology and Social Medicine, University of Antwerp, Antwerp, Belgium; 2 Department of Medical Microbiology, College of Medicine, Faculty of Health Sciences, University of Manitoba, Winnipeg, Manitoba, Canada; 3 Sexually Transmitted Infection Unit, Institute of Tropical Medicine, Antwerp, Belgium; 4 University of Cape Town, Cape Town, Western Cape, South Africa; 5 Department of Dermatology, Third Affiliated Hospital, Sun Yat-Sen University, Guangzhou, China; 6 National Institute for Communicable Diseases, Johannesburg, South Africa; 7 CBS-KNAW Fungal Biodiversity Centre, Utrecht, The Netherlands; 8 University of Alberta Microfungus Collection and Herbarium, Devonian Botanic Garden, Edmonton, Alberta, Canada; 9 Department of Dermatology, The Affiliated Hospital, Guizhou Medical University, Guiyang, China; 10 Cellular and Molecular Biology Unit, Corporación para Investigaciones Biológicas (CIB), Medellín, Colombia; 11 Institute of Biology, Universidad de Antioquia, Medellín, Colombia; 12 Broad Institute of Harvard and MIT, Cambridge, Massachusetts, United States of America; 13 Department of Microbiology, Stellenbosch University, Stellenbosch, Western Cape, South Africa; 14 Mycology Unit, Medical School & Pere Virgili Institute for Health Research, Universitat Rovira i Virgili, Reus, Spain; Duke University Medical Center, UNITED STATES

## Introduction

New species of *Emmonsia*-like fungi, with phylogenetic and clinical similarities to *Blastomyces* and *Histoplasma*, have emerged as causes of systemic human mycoses worldwide. They differ from classical *Emmonsia* species by producing a thermally-dependent, yeast-like phase rather than adiaspores, and by causing disseminated infections, predominantly in immunocompromised patients and often with high case-fatality rates. Such differences will be important for clinicians to consider in diagnosis and patient management, and for microbiologists who may encounter these fungi with increasing frequency.

## Adiaspiromycosis Is a Rare and Limited Disease in Humans

Until recently, the clinical relevance of the genus *Emmonsia* was limited to a very rare and unusual pulmonary disease named adiaspiromycosis, caused by two species, *Emmonsia crescens* and *Emmonsia parva*. The disease follows inhalation of aerosolized conidia, released from mycelia found in soil. In the lungs, the conidia undergo a dramatic enlargement, from ~2–4 μm to 40–500 μm in diameter—a volume increase of up to a million-fold [1]. Emmons and Jellison called these swollen cells adiaspores, from the Greek α– (not, without), –δια– (by, through), and –σπορα (seed, sowing), in reference to the fact that they neither replicate nor disseminate [1]. However, their presence in the host may provoke a foreign body reaction, resulting in granulomatous lung disease [2,3]. Disease severity is dependent on inoculum size and host response, with a spectrum ranging from subclinical pneumonia to diffuse pulmonary disease causing hypoxic respiratory failure and, occasionally, death [2–4].

**Fig 1:**
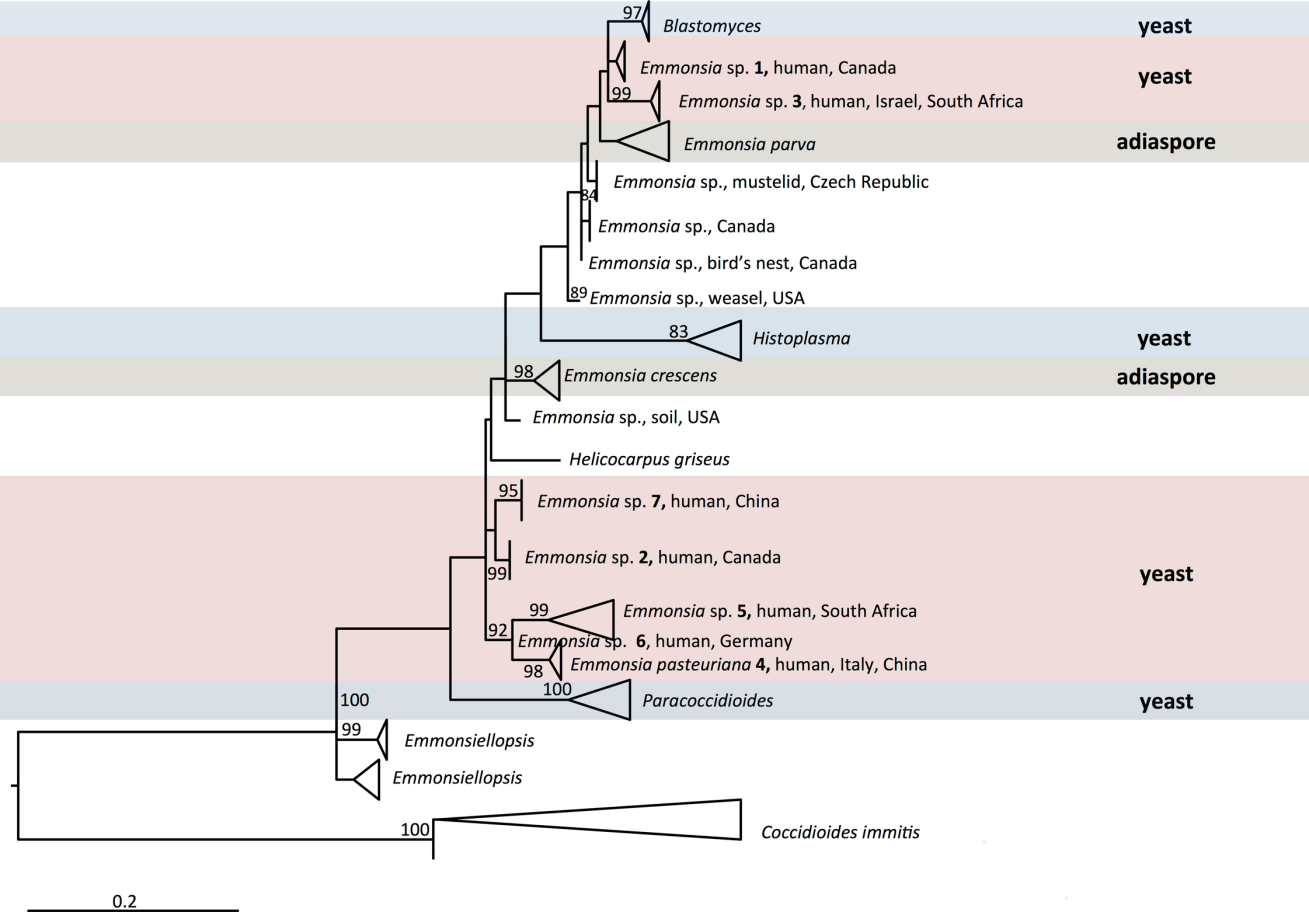
Maximum likelihood phylogeny inferred using RAxML v. 8.0.0 employing GTRCAT model and 1,000 bootstrap replicates. Bootstrap branch support above 80% is shown. Multiple sequences in the same species are collapsed. Genera included in the family Ajellomycetaceae are shown, with the exception of *Lacazia loboi*, which could not meaningfully be aligned.

Adiaspiromycosis is common in rodents and other small terrestrial mammals. For instance, nearly a third of wild British mammals sampled had signs of the disease [5]. *E*. *crescens* has been reported to cause adiaspiromycosis in over 118 mammalian species with a global distribution [2]. Sequencing data from some *E*. *parva*-like isolates from animals has implicated different, as-yet-undescribed species (e.g., *Emmonsia* sp. from weasels in the United States [6] and *Emmonsia* sp. from mustelid in the Czech Republic), which are included in the phylogenetic tree of Fig 1.

Occasionally, humans can also be affected. The first human case of adiaspiromycosis was reported in 1964 [7], and cases have since been reported worldwide [2,3]. *E*. *crescens* has been implicated in the vast majority of these infections. Unusual cases of infection by *E*. *parva* have also been reported in immunocompromised hosts [8,9]; however, clinical and histopathological findings were so atypical that, in the absence of molecular confirmation, the identification of the pathogen has been questioned [2,3]. Additionally, superficial adiaspiromycosis was reported to cause granulomatous conjunctivitis in 99 of 5,084 children (1.9%) screened in the Amazon basin, and histopathological examination of ocular nodules identified adiaspore-like structures in two of 14 cases [10]. The investigators identified diving in a nearby river as a risk factor, and surmised that conjunctival irritation from spicules of freshwater sponges provided a portal of entry. However, the identification of the putative pathogen remains unclear: the unusual exposure history and clinical features suggest that *Rhinosporidium seeberi* rather than *Emmonsia* species might have been involved.

## A Leap from Obscurity to Global Medical Importance

**Fig 2:**
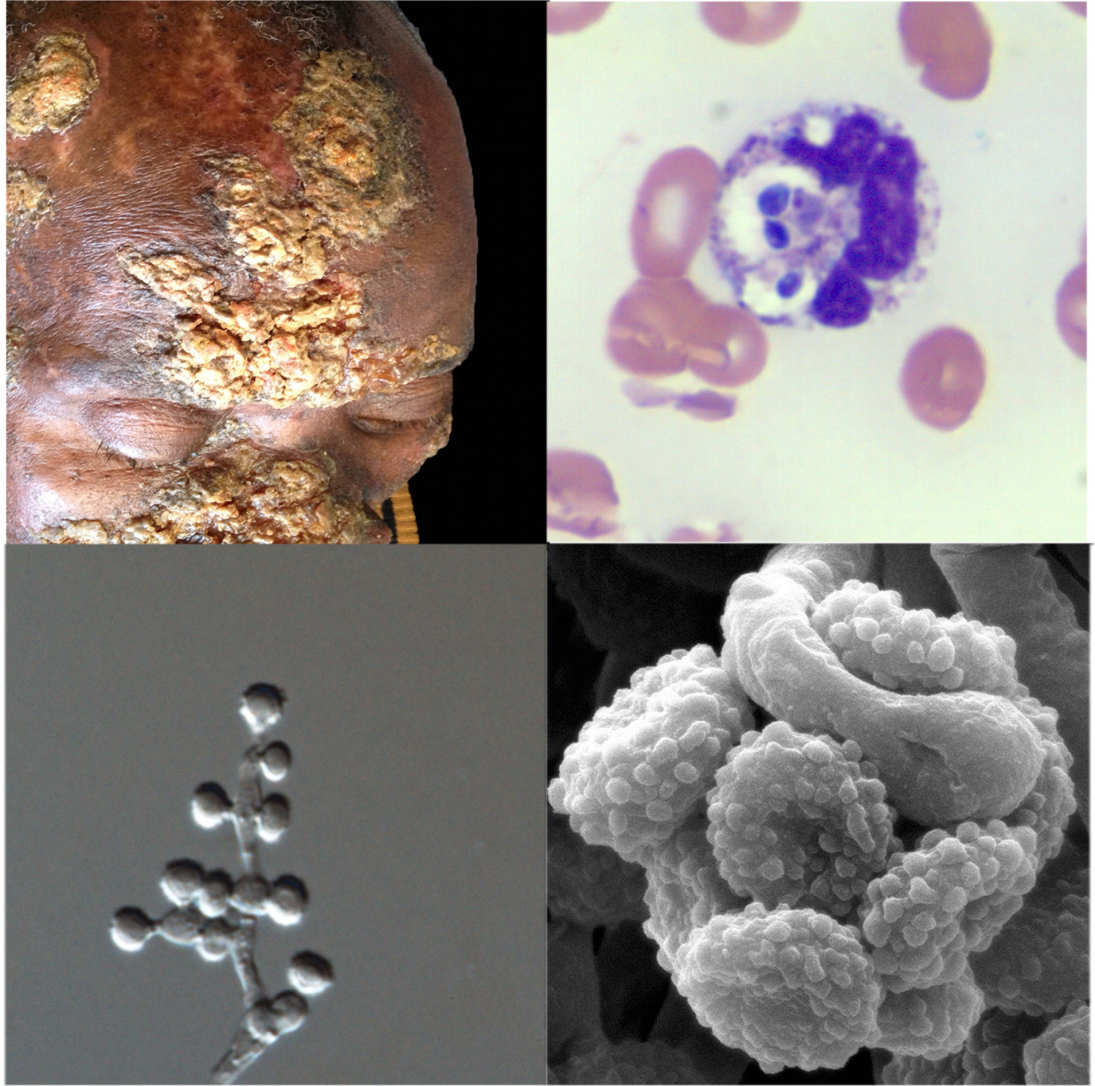
Clinical, pathological, and mycological facets of a novel *Emmonsia*-like fungus reported from South Africa. Top left: Hyperkeratotic skin lesions in a patient with disseminated *Emmonsia* disease (published with patient consent; courtesy of Dr. Tabie Greyling, Stellenbosch University). Top right: Peripheral blood smear showing neutrophils with multiple phagocytosed yeast-like cells (Wright-Giemsa staining x1,000). Bottom right: Electron microscopy image of conidia (courtesy of Dr. Monica Birkhead, National Institute of Communicable Diseases). Bottom left: Light microscopy image of conidiophores and conidia (x1,000).

**Fig 3:**
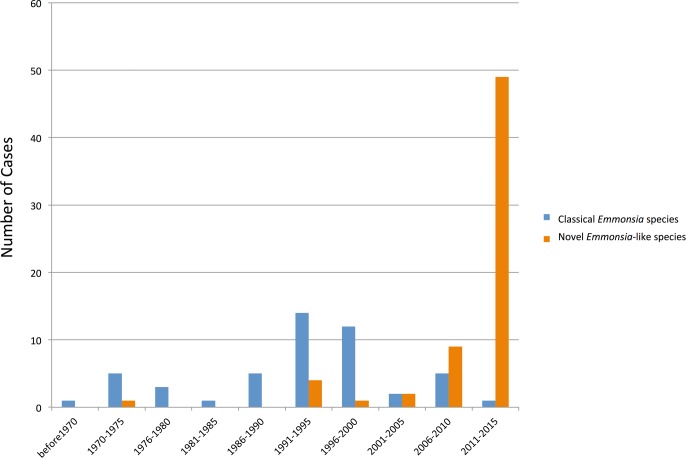
Timeline of human cases of disease caused by classical *Emmonsia* species (with adiaspores at 37°C) and novel *Emmonsia*-like species (with yeast cells at 37°C). Vertical bars represent number of cases during 5-year intervals, as determined by literature review. The increase in cases of diseases caused by novel *Emmonsia*-like species occurring between 2006 and 2015 is primarily driven by recognition of HIV-associated cases in South Africa following the introduction of molecular identification protocols for dimorphic fungal infections in 2008.

Over the last four decades, reports have emerged of patients with unusual mycoses: in the laboratory, cultures have isolated molds with asexual reproductive structures that resembled *Emmonsia*, but clinical and histopathological pictures were more compatible with blastomycosis or histoplasmosis than adiaspiromycosis (Fig 2). Molecular sequencing has since confirmed that these fungi belong to a cluster of novel *Emmonsia*-like species (Fig 1). It remains unclear whether this cluster of *Emmonsia-*like species only emerged recently as human pathogens, or whether previous infections were merely underestimated. Support for the latter hypothesis comes from South Africa, where the introduction of molecular identification tools resulted in a dramatic increase in the number of cases of disseminated *Emmonsia* disease, commensurate with a decline in the number of cases of confirmed histoplasmosis [11]. The timing of human cases is illustrated in Fig 3.

The earliest recorded case, from 1970, was reported as an unusual case of blastomycosis in a patient from Alberta, Canada, a region considered non-endemic for this disease [12]. The patient presented with neurological symptoms; at autopsy, histopathology sections revealed atypical yeast-like cells in lung and brain, and a fungus resembling *Blastomyces dermatitidis* was isolated from cerebral spinal fluid and lung. However, the organism failed to produce typical conidia or thick-walled yeast cells in culture [12]. Retrospective examination of the culture, including sequencing of large ribosomal subunit and internal transcribed spacers (ITS) loci, determined that this isolate represented a novel *Emmonsia*-like fungus (*Emmonsia* sp. 1, Fig 1) [6].

The next recorded case of disseminated infection, in 1992, was an HIV-infected male from Saskatchewan, Canada, with cutaneous lesions. Skin biopsy culture grew a mold resembling *Emmonsia*; retrospective genetic analysis demonstrated the novelty of this species (*Emmonsia* sp. 2, Fig 1) [6,13]. A second case from Saskatchewan occurred in 2003, in an Asian renal transplant recipient with atypical pneumonia. An *Emmonsia*-like fungus was cultured from blood and respiratory specimens [14].

A third novel *Emmonsia*-like fungus (*Emmonsia* sp. 3, Fig 1) was found in 1993, causing granulomatous mucocutaneous lesions in an immunocompetent man in Israel [13]. The yeast-like cells seen on skin biopsy were originally thought consistent with blastomycosis [13], but the fungal cells were smaller, and an *Emmonsia*-like fungus was isolated [15]. A second case of infection was recently identified in an immunocompetent South African patient with a mycotic brain abscess [16]. Histopathology of brain biopsy was suggestive of blastomycosis [17], but the ITS sequence of an *Emmonsia*-like fungus isolated from brain tissue was nearly identical to that of the fungus cultured from the Israeli case [18], suggesting these isolates belong to the same species.

In 1994, an HIV-infected woman from Italy was diagnosed with a disseminated mycosis [19]. Biopsies of cutaneous lesions revealed small, yeast-like cells in tissue, and a thermally dimorphic *Emmonsia*-like fungus was isolated in culture [19]. This was described as the novel species *E*. *pasteuriana* (*E*. *pasteuriana* 4, Fig 1) [20]. An additional case was reported in 2011, in a liver transplant recipient with HIV infection from Spain, with pulmonary and cutaneous lesions [21,22]. Recently, two additional cases of *E*. *pasteuriana* were reported from Guangzhou, China, in a renal transplant recipient [23] and in a patient treated with high-dose corticosteroids [24].

In 1995, a fifth novel *Emmonsia*-like fungus (*Emmonsia* sp. 5, Fig 1) was isolated from a skin biopsy of an HIV-infected man in South Africa with widespread skin lesions [11]. The fungus had yeast-like structures instead of adiaspores. Another case was not identified until 2008, when routine molecular identification of dimorphic fungi was adopted at several microbiology laboratories in South Africa. Over the next 3 years, 13 cases of disseminated disease were diagnosed among HIV-infected adults [11]. By 2015, 55 cases had been diagnosed [17,25]. Among these, 53 were HIV-infected, and another was a renal transplant recipient. Pulmonary disease was common and cutaneous involvement near universal. The case fatality rate was 48% [17].

In 2000, a sixth novel *Emmonsia*-like fungus (*Emmonsia* sp. 6, Fig 1) was implicated as the cause of isolated, necrotizing pneumonia in a German farmer with rheumatoid arthritis treated with corticosteroids. Yeast-like cells were present in a transbronchial biopsy [26]. And finally, in 2005, infection due to a seventh novel *Emmonsia*-like fungus (*Emmonsia* sp. 7, Fig 1) occurred in a diabetic patient from Beijing, China [27], who had pulmonary and cutaneous disease.

## Key Emergent Traits of Disseminated *Emmonsia* Disease

The newly recognized *Emmonsia* species differ significantly from the agents of adiaspiromycosis, most notably in displaying thermal dimorphism, and growing as a mold at 25°C and as yeast-like cells (rather than producing adiaspores) at 37°C. Consequently, major differences exist in the pathogenesis, epidemiology, disease spectrum, diagnostic findings, course, and management.

The primary route of infection, conserved among *Emmonsia* spp., is presumed to be inhalation of airborne conidia released from saprophytic mycelia in soil [2]. Similarities in pathogenesis between classical and emerging *Emmonsia*-like species end there. Once in the mammalian (human) host tissue, rather than swelling to sterile, static adiaspores, conidia of newer *Emmonsia*-like species convert to yeast-like cells capable of replication and extra-pulmonary dissemination. Disease results from tissue invasion [28], although a contribution of host response to pathogenesis is suggested by apparent unmasking immune reconstitution inflammatory syndrome in some HIV-infected patients who develop stigmata of disease upon initiating antiretroviral treatment [17].

Most reported patients with adiaspiromycosis have been immunocompetent [2,4], although exceptions exist [3,4]. In contrast, nearly all reported patients with disease due to emerging *Emmonsia*-like species have had profound impairment of cell-mediated immunity. These have included HIV infection in the vast majority (among whom the median CD4^+^ T-lymphocyte count was 16 cells/μL) [11,17,19,21,25]. Other associated conditions include solid organ transplantation [14,16,21,23] and corticosteroid use [23,26].

Results of histopathological and microbiological investigations of adiaspiromycosis and disseminated *Emmonsia* disease are unmistakably different. The *sine qua non* of adiaspiromycosis is the presence of adiaspores in tissue; the causative agents have rarely been cultured from humans [2,13]. On the other hand, the histopathological hallmark of disseminated *Emmonsia* disease is yeast-like cells in tissue [11,12,15,17,19], which may be mistaken for *Histoplasma* [17] or *Blastomyces* [12,15,17]. Fungi may be isolated from clinical specimens, particularly with prolonged incubation on routine fungal culture media [2,12,14,17,19,21,23,25,26].

The natural histories of adiaspiromycosis and disseminated *Emmonsia* disease are quite different and, consequently, principles of management differ, although evidence to guide therapy is anecdotal. Adiaspiromycosis is generally self-limiting, and fatalities are exceptional [29–31]. Because this disease results from host response, corticosteroids have been advocated in severe cases; the role of antifungals remains uncertain [3,31,32]. In contrast, disseminated *Emmonsia* disease appears to be a progressive disease in many patients, particularly immunocompromised hosts, in whom case-fatality rates approach 50%; among these patients, antifungals appear to be imperative [17].

## Disentangling the Ajellomycetaceae

Even before the discovery of newer *Emmonsia* spp., the taxonomy of these species was a matter of debate among mycologists [2,33]. The sexual stage (teleomorph) of *E*. *crescens* belongs to *Ajellomyces*, the same genus as the teleomorphs of *B*. *dermatitidis* and *H*. *capsulatum* [34], suggesting a close relatedness. Genetic studies have also demonstrated a close phylogenetic relationship between *Emmonsia* and *Blastomyces* [13,35]; in fact, *E*. *parva* is more closely related to *Blastomyces* than to *E*. *crescens* [13]. Members of both genera produce budding, yeast-like cells and share genetic similarities, perhaps even justifying the assignment of *Blastomyces* and *Emmonsia* species to a single genus [13,35,36]. Full genome sequencing of multiple isolates of all *Emmonsia*-like species will provide greater resolution of phylogenetic relationships, and may help to clarify taxonomic boundaries. Recently, genome sequences for two animal-associated *Emmonsia* strains were made publicly available in NCBI (isolated from lungs of a rodent and from a weasel, and catalogued under Bioprojects PRJNA178252 and PRJNA178178, respectively), and submissions are in progress for additional human-associated, pathogenic strains shown in Fig 1. Preliminary phylogenetic analyses suggest that most of the new human-associated *Emmonsia*-like fungi form a single, derived clade in the Ajellomycetaceae, while agents of adiaspiromycosis appear to be polyphyletic (Fig 1).
